# From narratives to numbers and back: Assessing the psychosocial aspects of diabetes in the era of high technology with emerging qualitative and quantitative methodologies

**DOI:** 10.1111/dme.70206

**Published:** 2026-01-02

**Authors:** Dominic Ehrmann, Eloise Litterbach, Sonya Deschenes, Rita Forde, Norbert Hermanns, Maaike Horsselenberg, Mandy Jansen, Amy McInerney, Eimear Morrissey, Andreas Schmitt, Uffe Søholm, Giesje Nefs

**Affiliations:** ^1^ Research Institute Diabetes Academy Mergentheim (FIDAM) Bad Mergentheim Germany; ^2^ Department of Clinical Psychology and Psychotherapy University of Bamberg Bamberg Germany; ^3^ German Center for Diabetes Research (DZD) München‐Neuherberg Germany; ^4^ School of Psychology | Institute for Health Transformation Deakin University Geelong Victoria Australia; ^5^ The Australian Centre for Behavioural Research in Diabetes, Diabetes Victoria Carlton Victoria Australia; ^6^ School of Psychology University College Dublin Dublin Ireland; ^7^ School of Nursing and Midwifery University College Cork Cork Ireland; ^8^ Department of Medical Psychology Radboud University Medical Center Nijmegen the Netherlands; ^9^ CWZ Academy, Canisius‐Wilhelmina Hospital Nijmegen the Netherlands; ^10^ Center for Pediatric and Adolescent Diabetes, Vivendia Nijmegen the Netherlands; ^11^ Department of Population‐Based Medicine University of Tuebingen Tübingen Germany; ^12^ Centre for Health Research Methodology, School of Nursing and Midwifery University of Galway Galway Ireland; ^13^ Diabeter, Center for Focused Diabetes Care and Research Rotterdam the Netherlands; ^14^ Department of Medical and Clinical Psychology, CoRPS—Center of Research on Psychological Disorders and Somatic Diseases Tilburg University Tilburg the Netherlands; ^15^ Diabeter Centrum Amsterdam Amsterdam the Netherlands

**Keywords:** mixed methods, psychosocial aspects, qualitative research, quantitative research

## Abstract

**Aims:**

Rapid changes in diabetes therapy combined with limitations of traditional methodological approaches challenge the field of psychosocial research to adequately capture the experiences of people with diabetes. This narrative review provides an overview of emerging qualitative and quantitative approaches that can advance the study of psychosocial aspects of diabetes.

**Methods:**

We searched PubMed and Google Scholar for English‐language articles regarding novel qualitative and quantitative methodologies.

**Results:**

Emerging qualitative methodologies aim to increase the transferability of lived experiences to other contexts and populations by employing novel ways to stimulate interactions and using digital tools. Culturally sensitive methods (e.g. yarning) and the use of pictures (e.g. photovoice) and storytelling methods (e.g. story completion) can capture more diverse experiences and sensitive topics while being able to minimise social desirability. Online qualitative surveys can increase the reach while artificial intelligence (AI) can be implemented in qualitative research protocols. Emerging quantitative methodologies aim to better understand dynamic within‐person processes. With repeated daily smartphone‐based assessments (e.g. ecological momentary assessment) and passive sensor‐based data collections (e.g. digital phenotyping), intensive longitudinal data can be collected that allow for n‐of‐1 trials, especially in combination with continuous glucose monitoring. Quantitative data can also be used to identify clusters/subgroups of people with shared experiences. Innovative digital twin technology and AI offer intriguing possibilities that can advance the field towards precision mental health care.

**Conclusions:**

Several innovative methodologies (will) enrich our understanding of psychosocial aspects in diabetes. To fully capitalise on these methodologies, co‐design and mixed methods approaches are necessary.

## Graphical abstract

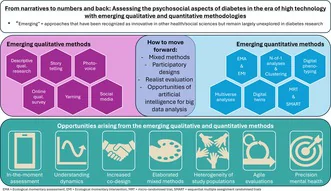

**What's new?**

- Diabetes management has substantially changed due to technological, pharmacological, and digital innovations. Novel qualitative and quantitative psychosocial methodologies are needed to mirror these advances in diabetes management and care.
- Emerging qualitative methodologies seek to enhance the transferability of lived experiences across contexts and populations by introducing innovative interaction formats (e.g. yarning, photovoice, storytelling, and online surveys) and leveraging digital technologies.
- Emerging quantitative methodologies use intensive longitudinal data from smartphone‐based assessments, passive sensors, and continuous glucose monitoring to model within‐person dynamics.

## 1 INTRODUCTION: WHY DOES THIS ISSUE NEED TO BE ADDRESSED NOW?

Diabetes research has largely focused on biomedical outcomes, yet the psychosocial aspects of living with diabetes are equally important.^1^ Supporting psychosocial factors including mental health and wellbeing is essential for overall health, optimal glucose levels, fewer complications and effective self‐management.^1^, ^2^ Thus, psychosocial aspects have taken a firm place in diabetology due to thirty plus years of research.^3^, ^4^, ^5^, ^6^, ^7^, ^8^ Their clinical importance, as well as the call for their monitoring and screening, has been emphasised in several key care guidelines.^1^, ^2^, ^9^, ^10^, ^11^ Furthermore, questionnaire‐based person‐reported outcome measures (PROMs) are now more commonly included in clinical trials.^12^, ^13^, ^14^

To further understand psychosocial aspects, it must be considered that diabetes therapy has dramatically changed over the last 10 years and will continue to change due to technological (e.g. continuous glucose monitoring [CGM], automated insulin delivery [AID]),^15^ pharmacological (e.g. incretin‐based therapies)^16^, ^17^ and artificial intelligence^18^, ^19^ innovations. New therapies are on the horizon such as immunotherapies^20^ and stem cell therapies^21^ that will have an impact on people with diabetes in ways that we have not even begun to understand.^22^, ^23^

There is a general expectation that new therapies will change the lives of people with diabetes. While positive psychosocial experiences of, for example new technologies are indeed known from clinical practice, qualitative research and cross‐sectional surveys,^12^, ^24^, ^25^ they are often not reflected in trial‐based PROM change with much smaller (or even insignificant) effect sizes compared to glycaemic parameters.^24^, ^26^, ^27^, ^28^, ^29^ This begets the question of whether our existing psychosocial methods are suited to adequately capture the dramatic changes in diabetes therapy. If corresponding effects of new therapies on PROMs cannot be shown, there is the risk that psychosocial research will be devalued. This pattern is already visible in recent CGM and AID publications, in which PROMs are typically reported only in supplementary material due to insignificant findings.^30^, ^31^, ^32^

Whether our current methodological approaches in diabetes psychosocial research, largely developed when diabetes therapy was rather analogue, are still suited to capture the impact of modern therapy remains open to question. The fields of health psychology and behavioural medicine must mirror advances in diabetes management and care with new methodologies that reflect daily living with diabetes and are important to people with diabetes and, as such, are based on the lived experiences.

In general, development, evaluation and implementation of traditional PROMs often lag the pace of therapeutic innovations. Digital technology can offer new ways to conduct qualitative and quantitative research with and on PROs in more agile ways. In addition, emerging qualitative and quantitative research may provide new insight into why associations between PROs and medical outcomes are small/moderate and may offer a more in‐depth understanding of the intricate relationship between psychosocial and medical outcomes.

This narrative review provides an overview of emerging qualitative and quantitative approaches with high potential to further advance the study of the psychosocial aspects of diabetes. We searched PubMed and Google Scholar for English‐language articles regarding novel qualitative and quantitative methodologies already applied in or potentially relevant for psychosocial diabetes research. For the purposes of this review, we defined ‘novel’ methodological approaches as those that have been recognized as innovative in other health or social science domains but remain largely unexplored in diabetes research.

## 2 WHERE ARE THE CURRENT KNOWLEDGE GAPS?

Table 1 highlights key limitations of traditional qualitative and quantitative research methodologies and offers a rationale how and why current methodological approaches may fall short in assessing newer diabetes therapies.

**TABLE 1:** Key limitations of traditional qualitative and quantitative research methodologies and resulting gaps in psychosocial diabetes research.

	Limitation	Elaboration	Gaps
a.	Retrospective questionnaires	Recall bias Only provides a sum score of past experiences → information on intensity only No/limited information on variability, frequency or duration of experiences	Gap: Real‐time assessment Due to CGM, increased focus on real‐time insight into glucose dynamics Lack of a time‐sensitive, real‐time assessment of PROs over a longer period in people's daily lives
b.	Lack of intraindividual dynamics and interindividual variability	Limited assessments with long inter‐measurement intervals of panel research No/limited information on dynamic processes unfolding in an individual No information on within‐person associations	Gap: Dynamics Better understanding of dynamic processes, particularly relating to the association of glucose levels and PROs Better understanding how different (groups of) people vary in these dynamic associations and processes
c.	Reliance on group‐level results	Between‐person differences do not necessarily reflect the experiences of individuals Can average out effects Rather small associations between PROs and medical outcomes	Gap: Precision mental health Better understanding how group‐level findings can be translated to the experiences of individuals unfolding in everyday life. Moving beyond group‐level findings and focusing on subgroup or individual‐level experiences Mirror the call for precision medicine by implementing precision mental health
d.	Sluggishness of evidence generation	Traditional approaches to evidence generation (RCT designs) take a long time Rigid design to test interventions Rigid design to develop and evaluate PROMs No/limited information about which aspects of a complex intervention work Questions around robustness of observational and clinical data to inform interventions	Gap: Agile evaluations Gap between the pace of innovations and the evidence for it (called the ‘Catch 22’ dilemma) Agile testing of complex interventions or stepped care approaches Agile development and validation of PROMs Robustness‐check regarding study design and/or analytical decisions (‘Multiverse analysis')
e.	Selectivity of interview data	Risk of over‐interpretation of the voices of those wanting/feeling able to share their experiences Resource intensive → Limited to a small number of participants	Gap: ‘Heterogeneity’ Underrepresentation of diverse lived experiences Transferability of lived experiences to other contexts and population groups Effective ways to conduct, code and analyse rich qualitative data
f.	Top‐down design	Qualitative and quantitative studies often solely designed by researchers/clinicians No/limited/tokenistic participation of people with lived experience	Gap: Co‐design Participatory approaches Co‐design of research
g.	Separation of quantitative and qualitative methods	No/limited integration of qualitative methods and insights into quantitative designs No/limited integration of quantitative methods and insights into qualitative designs	Gap: Mixed methods Bidirectional stimulation between qualitative and quantitative methods Equality of qualitative and quantitative approaches Fostering of mixed method approaches

Abbreviations: CGM, continuous glucose monitoring; PROs, person‐reported outcomes; RCT, randomised controlled trial.

Qualitative research methods have been laying the foundations for understanding psychosocial aspects of diabetes for almost 100 years.^33^ Though underutilised and underrepresented in diabetes research,^34^ they offer context and nuance to complement quantitative research interpretation and translation.^35^ They enhance our understanding of the implications and complex psychosocial impacts of lived experiences,^36^ but can also promote shared experiences, self‐compassion and agency.^37^ Yet, traditional qualitative data collection approaches (e.g. interviews, focus groups) have their limitations.^38^, ^39^ These include an underrepresentation of the voices of people who feel reluctant or not able to share experiences in face‐to‐face vocal research, limitations to the number of participants who can feasibly take part due to the intensity of data collection and analysis and sub‐optimal reporting of the researchers' background and reflexivity processes.

Traditional quantitative psychosocial research in diabetes focuses on questionnaires using a retrospective self‐report approach often deployed in clinical trials, cross‐sectional research or panel research with limited assessments and long inter‐measurement intervals.^12^, ^13^ This research inherently fails to capture dynamic processes as they actually unfold in an individual because changes and associations within a person cannot be separated from the group‐level (between‐person) differences.^40^, ^41^ This means that findings that apply across many individuals do not necessarily hold true for the experiences of an individual over time. Thus, the translation of these group‐level data to the individual and therefore also to clinical practice is limited.^41^ In addition, commonplace questionnaires offering a retrospective sum score of experiences in the past days or weeks cannot account for the contextual dynamics of experiences, often suffer from recall bias, and can be influenced by the person's current emotional state.^42^, ^43^ Furthermore, ‘gold standard’ evaluation studies including randomised controlled trials (RCTs) often do not match the speed of innovations, thereby creating a ‘Catch 22’ dilemma of either lagging evaluation studies or use of untested interventions.^44^

## 3 EMERGING METHODOLOGIES IN QUALITATIVE RESEARCH: CAPTURING DIVERSE PERSPECTIVES

Qualitative methods have been engaged in designing relevant and community‐informed tools to measure individual and population level psychological and behavioural concepts. For example, traditional qualitative explorations of the social experiences of people with diabetes have been employed to inform the development of the diabetes stigma assessment scales,^37^, ^45^ as well as person‐centred core outcome sets.^46^ Qualitative longitudinal research is innovative in diabetes studies because it traces experiences over time, revealing how self‐management, health care relationships and barriers evolve.^47^, ^48^, ^49^, ^50^ For example, studies have shown that people's approaches to diet, medication taking and engagement with health services are not static but shaped by ongoing life events, family responsibilities and broader structural factors. Qualitative longitudinal research provides unique insights into the temporal dimensions of living with diabetes, including how barriers and facilitators to care may shift across time.

More recently, emerging qualitative methods have been employed, such as qualitative co‐design to develop a stigma tool for gestational diabetes mellitus (GDM)^37^ and qualitative analysis of online lived experience blogs to review the content validity of existing tools [S1].

Though qualitative research is time intensive, advancing methodologies are exploring more efficient ways to capture diverse lived experiences and help bridge knowledge gaps. Previous qualitative work has captured complex barriers and enablers of diabetes self‐management [S2–S6]. More recently, novel ways to capture lived experience narratives, such as storytelling and yarning, are helping to enhance our understanding of community experiences, needs and preferences.

### 3.1 Story telling methods

Beyond traditional semi‐structured or structured qualitative interviewing, innovative story telling methods have been engaged to gather people's diabetes experiences. Group‐based story completion, where people share their own stories, prompted by choosing a ‘story stem’, in small group settings, has shown to facilitate self‐reflection and meaning making of one's own experience, as well as collaborative learning about how to best support and engage in diabetes self‐management [S7]. In the health care context, story completion is thought to minimise social desirability bias and be appropriate for exploring sensitive topics, as it allows the participant to engage as a third party (for example, playing the role of another person outlined in the story stem) [S8]. Story completion methods may offer improved capacity for exploring sensitive psychosocial aspects of diabetes, such as stigma and eating disorders.

### 3.2 A culturally specific approach: Yarning

Group‐based ‘yarning’ is also an alternative to traditional inquiry in which researchers and participants engage in a respectful, reciprocal ‘yarn’ (i.e. conversation) to share stories and create new knowledge. Though not a new concept, yarning has become increasingly understood as a culturally appropriate way to understand experiences of Australian Indigenous People supporting culturally safe care and reducing miscommunication in treatment settings [S9, S10]. Specifically, yarning circles led by Indigenous People have helped us to understand the ongoing impact of colonisation and systemic inequities as a barrier to diabetes care in Australia [S11]. These opportunities for people with diabetes to come together as a community and narrate their story in their own way are an opportunity for improved understanding of experiences and health care needs as well as informing more responsive models of care [S11]. Beyond diabetes, yarning has been applied to mental health research, maternal health and health workforce education, demonstrating its versatility in addressing diverse health challenges [S12]. Yarning represents both a methodological innovation and a culturally respectful practice that enhances understanding of health experiences and supports more equitable health care delivery [S13].

### 3.3 Digitalisation of qualitative data

Video technology has transformed the way we conduct qualitative research which goes beyond traditional in‐person or telephone interviews and focus groups [S14]. Using video communication platforms to speak with people living with diabetes, caregivers, health professionals and stakeholders has enabled high quality and acceptable qualitative data collection [S14]. Through online video interviewing, we have the capacity to reach diverse and widespread geographical areas, and when compared to using audio alone, we can still develop rapport, understand emotions using body language and social cues and interview with empathy. This not only widens the pool of participants as it can remove traditional barriers (e.g. needing to travel to a study site) but also increases our capacity to safely explore sensitive topics as well as improve the research participation experience for people with diabetes. Video technology further enhances research capacity when secure options for automated transcription can be enabled, which reduces financial and time costs.

Online qualitative surveys also allow the collection of large‐scale qualitative information. This method has been increasing in psychosocial diabetes research [S15, S16]. It includes both mixed methods and free‐text‐only opportunities to provide descriptions of diabetes‐related experiences, perceptions, preferences and impacts. Given that qualitative data collection and analysis can be time and resource intensive, sample sizes tend to be smaller and more homogenous than quantitative samples. Large pools of qualitative responses to questions can unpack phenomena within more diverse samples [S15]. These large‐scale findings tend to be more transferable to the broader population. While online qualitative surveys may be limited in depth and emotion, with no probing opportunities, describing lived experiences on such a large scale has been critical for understanding #LanguageMatters and health care interactions on a broader scale [S2, S5]. With large‐scale qualitative data, AI may be able to complement existing analysis techniques for textual interview transcriptions during early stages of analysis [S17]. While AI cannot replace the expertise of a skilled qualitative research team in understanding context and nuances, AI may have the capacity to support qualitative analysis during data organisation and initial coding framework development.

### 3.4 Photovoice: A picture says more than a thousand words

Photovoice techniques are being used to support shared dialogue and meaning making of diabetes experience(s) [S18]. Photovoice methods include using photographs, pictures, drawings or videos created by the participant as data. Images are often accompanied by discussion or explanation, using multiple senses to verbally and visually explore complex phenomena. Such methods can enhance meaningful involvement and support community empowerment by giving autonomy to the participant to tell their own story [S18]. They are ideal for communication between researchers and participants who do not speak the same native language, people who have lower literacy levels or have trouble articulating ideas [S18] and underserved populations [S19, S20]. Further advances of photovoice techniques include arts‐based film making to unpack and empower advocacy around sociocultural problems (e.g. diabetes stigma) [S20] and using art for young people to express their emotions surrounding and experiences of living with diabetes [S21].

### 3.5 Social media

In understanding diverse experiences of people living with, caring for a person with or being at increased risk of diabetes, social media offers opportunities for observing publicly available, real life, online social interactions [S22]. Passively observing social media interactions and narratives can offer authentic insights around (lived and constructed) experiences and support seeking behaviours [S23], as an alternative to real time direct observation and qualitative interviewing, which can be time intensive and risk social desirability bias. However, such research involves post hoc analyses, which cannot be queried for accuracy or clarification. Further, concerns for genuinity of online descriptions have been raised [S24] because of the nature of online personas (i.e. who you are online) which may differ from actual experiences [S25]. Social media can also be used to observe and understand sensitive topics which may carry stigma or be difficult to disclose to a researcher or health professional in‐person, but that people would more willingly share in an online setting. For example, exploring first person written experiences of diabulimia posted in blogs, researchers have gained insights into perceptions and beliefs around eating disorders in diabetes and the use of insulin to manage weight outcomes [S26]. Further research could explore public chat forums, such as open Facebook groups and Reddit Communities, the presence of and engagement with social media influencers, and messaging, language and representations of psychosocial topics in diabetes. Important ethical considerations of this method include how to negotiate informed consent and appropriate reporting [S27].

### 3.6 Descriptive qualitative research: Assessing the who, what and where

Descriptive qualitative research is a methodological approach focused on accurately and systematically describing the ‘who, what, and where’ of a phenomenon from a participant's subjective perspective, aiming for a factual account of experiences or events without deep theoretical abstraction or explanation [S28]. This might involve asking people with diabetes straightforward questions such as ‘What challenges did you face when starting insulin pump therapy?’ or ‘How do you decide when to measure your glucose during the day?’ [S29, S30]. The emphasis is on capturing participants' accounts of their experiences in their own words, to provide a clear description of barriers, facilitators and contextual factors in diabetes care, rather than interpreting these accounts through a specific theoretical lens. Descriptive qualitative data could also be paving the way for designing, implementing and evaluating diabetes awareness and related risk‐reduction campaigns, programs and policies, ensuring they are community‐informed, relevant, acceptable and tailored. To date, there is little qualitative evidence describing or evaluating perceptions of risk awareness or reduction campaigns. A recent evaluation of archival type 2 diabetes risk‐reduction campaign videos in Australia between 2005 and 2015 showed the videos to be stigmatising to many and had little positive influence on diabetes attitudes, motivation or self‐efficacy [S31]. Findings indicate that the messaging must be informed by the community it is intended for to develop ethical diabetes‐related risk messaging that does not unintentionally cause harm [S31, S32].

## 4 EMERGING METHODOLOGIES IN QUANTITATIVE RESEARCH: ZOOMING IN ON THE INDIVIDUAL

CGM made it possible to track self‐care in real time, highlighting glucose variability and more detailed glucose management [S33].^37^ Emerging quantitative methodologies, including ecological momentary assessment (EMA), digital phenotyping and n‐of‐1 trials, try to mirror such advancements by capturing daily life dynamics and assessing PROs with greater temporal resolution. Theories emphasising dynamics and feedback loops have begun to directly connect to emerging methodological approaches. For example, in relation to mental health development, Wichers posits that momentary experiences and behaviours act as building blocks of mental health symptom development, and Borsboom suggests that mental health conditions emerge from interactions among individual symptoms, supporting EMA, digital phenotyping, symptom‐level approaches [S34–S36].

These novel approaches impact (a) the type of insights that can be generated, (b) the development of new intervention strategies and (c) the evaluation of the impact of novel insights and interventions.

### 4.1 Insights: Methodologies to provide a more detailed understanding

#### 4.1.1 Ecological momentary assessment

Intensive longitudinal data methods comprise several ambulatory assessment methods such as EMA, experience sampling and daily diaries that allow for the repeated sampling of PROs in participants' daily life, usually via a smartphone [S37].^43^ It aims at assessing experiences, attitudes and behaviours with their dynamics in real time (or close to real time) in daily life [S37].^43^ Instead of asking, for example how someone has been feeling over the last 2 weeks with a questionnaire, EMA can zoom in to momentary experience and ask, for example how someone is feeling right now or within the last hour(s). It thereby minimises recall bias and provides extended information besides an overall score such as the intensity, duration, variability and real‐life context of experiences and behaviours. Thus, with EMA, new PROs can be developed that reflect the day‐to‐day variability in diabetes management and related experiences. For example, the outcome Time with Distress was developed, defined as the percentage of days a person spent with high distress. This outcome examines each day separately and offers a measure of intensity and duration of diabetes distress [S38].

For psychosocial diabetes research, EMA is to questionnaires as CGM is to HbA_1c_. The combination of EMA and CGM enables the collection of high‐resolution, time‐sensitive data that captures both within‐person dynamics and between‐person associations or differences in glucose management and related psychosocial variables [S39, S40]. Within‐person associations examine how two variables co‐vary over time within the same individual (e.g. whether on days with higher glucose a given person also reports worse mood). Between‐person associations use aggregated averages across individuals (e.g. whether people who spend more Time in Range over 14 days also report higher average mood). Thus, within‐person and between‐person associations can lead to different conclusions: the relationship that holds within individuals over time does not necessarily reflect how people differ from one another at the group level. The integration of CGM and EMA also allows for the analysis of contemporaneous (i.e. assessed at the same time) and lagged (i.e. one assessment preceding the other) associations between CGM metrics and PROs, offering a detailed and personalised understanding of their daily interplay and paving the way for precision monitoring in diabetes care [S40].

Recent EMA studies have demonstrated that glucose values—especially Time in Range (TIR), Time Above Range (TAR) and Time Below Range (TBR)—are associated with same‐day and next‐day mood, stress and cognitive functioning [S38, S41–S43]. For example, higher TIR has been linked to improved evening mood, while TAR, particularly values above 250 mg/dL, has been associated with greater distress and more negative affect [S41, S44]. Zooming in at more timely effects of glucose on PROs, a recent study demonstrated that high and low glucose levels in the 120 minutes prior to EMA symptom reports were significantly associated with the intensity of diabetes‐related hypo‐ and hyperglycaemia symptoms [S45]. Notably, however, elevated baseline levels of depressive symptoms and diabetes distress significantly increased symptom reporting of nearly all of the 25 assessed symptoms, underscoring the amplifying role of mental health in the subjective experience of diabetes [S45].

EMA research has also demonstrated that nocturnal glucose dynamics appear to have a strong impact on next‐day functioning [S42, S46, S47]. Hypoglycaemia during the night has been associated with reduced cognitive performance [S43, S46, S47], energy and mood [S46] the next day. Interestingly, in the study of Søholm et al., self‐reported (subjective) hypoglycaemia showed stronger associations with psychosocial outcomes than sensor‐detected (objective) events, underlining the relevance of perception in shaping experience [S46]. Even the prevention of hypo episodes was associated with worsening of daily functioning, underscoring the need to capture person‐reported episodes, as prevented episodes are not ‘logged’ by CGM.

The combination of EMA and CGM also allows for a distinction between the experience and the physiology of glucose levels. Two recent studies have found that subjective perceptions of glucose levels significantly influenced mood, distress and sleep quality, while objective CGM‐based glucose levels did not [S46, S48].

#### 4.1.2 Intensive longitudinal data and N‐of‐1 analyses

Studies with intensive longitudinal data offer high‐density data per person from many repeated assessments for each person [S49].^40^ Thus, they can overcome the previously mentioned limitations of cross‐sectional and panel studies.^40^ When a sufficient number of data points are available for one person, idiosyncratic associations can be analysed within a N‐of‐1 framework. This approach allows the analysis of dynamic processes unfolding over time in a specific person^40^, ^41^; for example how glucose values of a person on 1 day are associated with mood on the next. Additionally, when intensive longitudinal data are collected from many persons, they can be analysed to see whether these within‐person associations can be generalised to the whole sample, or whether there are between‐person differences in these associations; for example if a demographic variable moderates the glucose‐mood association. Analysing between‐person differences in within‐person dynamics has the potential to identify subgroups or clusters of people for which a specific association is, for example stronger or weaker.

Diabetes is a condition that is well suited for intensive longitudinal data approaches, as CGM provides routinely collected, high‐frequency glucose measurements. Paired with EMA, the intra‐ and interpersonal dynamics between psychosocial factors and glucose regulation can be analysed. Examples of psychosocial diabetes research utilising intensive longitudinal data already exist [S43, S45, S46, S48, S50, S51] and first evidence of N‐of‐1 analyses in diabetes demonstrates the usefulness in better understanding the individual drivers of diabetes distress [S48] and individual associations of glucose and the experience of diabetes symptoms [S45].

#### 4.1.3 Digital phenotyping

Capturing a person's lived experiences in situ via data from their personal smartphones or wearables thereby quantifying their phenotype is called digital phenotyping and presents a relatively novel approach to study psychosocial and behavioural aspects of diabetes [S52]. For this digital phenotyping, intensive longitudinal data from EMA and CGM studies (active assessment) can be enriched with passively collected mobile sensing data from wearables and smartphone sensors (e.g. call and text logs, step count, geopositioning, screen time) [S52]. This technology allows for a shift from macro‐level, retrospective static assessments to a more precise quantification of everyday experiences and behaviours at the micro‐level. Capable of quantifying behavioural patterns such as exercise and movement patterns, mobile sensing may provide additional insight into the day‐to‐day impact of diabetes on mental health and behaviour in real time.

For example, with a combination of passive mobile sensing data and active meal logging in type 2 diabetes, Pai et al. demonstrated that each 1000 steps after a meal significantly decreased 3‐h postprandial glucose by 641 min mg/dL and that with every 100 kcal more, the postprandial excursions increased by 183 min mg/dL [S53]. Evidence for psychosocial digital phenotyping in diabetes is currently limited [S54], but two qualitative studies show high acceptance and feasibility of such solutions in people with type 1 and type 2 diabetes [S55, S56]. In the Smartphone, Behaviour and Mood study, the aim was to identify digital phenotypes of distress and the processes leading to distress in type 2 diabetes. McInerney et al. found high tolerability and high perceived benefits from this approach in 68 participants after 2 months of intensive digital phenotyping [S55]. Aguirre Vergara et al. reported insights from the PsyVoice study that aimed at identifying vocal biomarkers of diabetes distress and found positive attitudes towards voice‐based assistance in the management of diabetes distress [S56].

When combined with AI, digital phenotyping offers substantial potential to uncover associations and patterns between psychosocial and sensor‐derived variables that have eluded traditional statistical methods [S57].

#### 4.1.4 Cluster analysis

The above‐mentioned approaches deepen understanding of individual processes and can lead to personalised diabetes care. However, full personalisation is often not feasible or is associated with immense resources. A feasible option is to differentiate subgroups with shared characteristics (e.g. attitudes, emotions, behaviours) that can be targeted for intervention [S58–S60]. Methods to achieve this include a variety of cluster analysis techniques, most notably K‐means clustering and Latent Class/Profile analysis [S61, S62]. These techniques group individuals into a small number of interpretable clusters, which can be validated using demographic or clinical differences. The results from cluster analysis and cluster validation can be combined to create ‘personas’, that is subgroups with similar response patterns to guide targeted interventions [S63].

There are several examples of how cluster analyses have been applied across diabetes types [S58] and psychosocial domains. For example, among adults with type 1 diabetes from the U.S., cluster analysis identified five clinically meaningful clusters of readiness to adopt new diabetes technology [S63]. These ranged from ‘d‐Embracers’ (positive technology attitudes, low barriers to uptake) to people with response patterns indicating ‘high distress’ (most barriers to uptake). Similar studies have been undertaken among parents of children with type 1 diabetes [S64] and healthcare providers [S65].

### 4.2 Interventions: Towards more personalised treatment approaches

#### 4.2.1 Ecological momentary interventions

Insights about momentary dynamics in psychosocial variables (e.g. mood, diabetes distress) can be used to develop momentary interventions that target these dynamics in everyday life when they become problematic. Thus, the methodology of EMA can be refined to ecological momentary interventions (EMI) that offer real‐time personalised treatments through mobile technology [S66]. A promising EMI is just‐in‐time adaptive interventions (JITAIs) that offer interventions at the right moment, when a mental health issue emerges (just‐in‐time) and adapted to the situation and person (adaptive) in their everyday life [S67]. While JITAIs in diabetes exist that have proven effective [S68], their large‐scale implementation is still lacking [S69].

#### 4.2.2 Digital twins

An intriguing concept to advance personalised interventions towards precision medicine or precision mental health is that of digital twins. A digital twin is a virtual representation of a person that mirrors and models key features of a person based on past and real‐time data [S70–S72]. It allows the modelling of so‐called ‘what‐if’ scenarios. For example, digital twins can simulate *what* would happen to glucose management *if* certain treatment decisions were made. Based on this safe simulation, real‐world therapy decisions about the most promising intervention can be made. It therefore serves as a decision support system for clinicians but also for people with diabetes. In a landmark trial, Kovatchev et al. were the first to integrate a digital twin approach into an AID algorithm.^19^ In their study, people with type 1 diabetes could simulate what‐if scenarios replayed via their own digital twins that allowed them to experiment with how adjustments in basal and bolus settings would impact their glucose profile. This personalised feedback enabled them to make adjustments that led to a significant increase in time in range.

While a psychosocial digital twin is not yet established [S70], potential benefits for psychosocial care can be anticipated. For example, simulations could determine which type of psychosocial intervention would be most beneficial and assess how changes in self‐management behaviour, diabetes distress or sleep quality would affect glycaemic and psychosocial outcomes [S73].

### 4.3 Impact: Novel ways for clinical translation

#### 4.3.1 MRT and SMART

Micro‐randomised trials (MRTs) and sequential multiple assignment randomised trials (SMARTs) are innovative experimental designs designed to optimise adaptive intervention strategies such as EMIs and JITAIs. In MRTs, participants are repeatedly and randomly assigned to different intervention options (e.g. receiving or not receiving a digital prompt) at multiple decision points throughout the study [S74]. This high‐frequency randomisation allows researchers to assess the proximal, time‐specific effects of intervention components in real‐world contexts, providing granular evidence on when, where and for whom an intervention works best. In contrast, SMART designs focus on longer‐term adaptation by randomising participants to different intervention sequences over multiple stages [S75]. For example, individuals who do not show sufficient improvement after an initial treatment might be re‐randomised to either intensify the current approach or switch to an alternative. Both designs support the development of flexible, personalised strategies that reflect real‐world clinical decision‐making and the fluctuating psychosocial needs of people with diabetes, offering a more agile alternative to traditional RCTs, potentially addressing the ‘Catch 22’ dilemma of RCTs mentioned previously.

#### 4.3.2 Multiverse analyses

For study findings to influence clinical care, effects must be robust and reproducible. Robustness depends partly on study design and the many analytical decisions researchers make. Multiverse analyses can address this by testing a wide range of plausible analytic approaches for the same question, helping quantify uncertainty and the likelihood of replicating results [S76, S77]. By revealing which analytic or design choices most influence reproducibility, they can guide optimal methodology for future studies. For example, Niemeijer et al. analysed how the choice of sensor to detect sleep characteristics (e.g. accelerometer, light) impacted the prediction of sleep quality, negative affect and depression [S78]. For psychosocial diabetes research, such analyses could increase trust and translation to clinical care.

## 5 HOW TO MOVE FORWARD?

**FIGURE 1:**
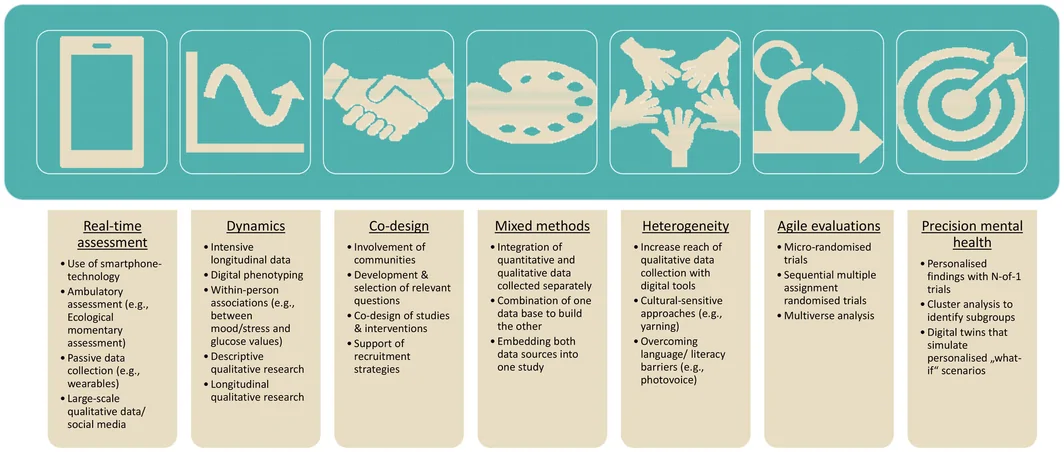
Fit of emerging qualitative and quantitative methodologies to key gaps in psychosocial diabetes research.

The above‐mentioned examples of novel approaches for generating and analysing qualitative and quantitative data show high potential in addressing current gaps of psychosocial diabetes research (Figure 1). To fully capitalise on these methodologies, we suggest an increased focus on the following overarching approaches.

### 5.1 Mixed method approaches

Mixed method research combines quantitative and qualitative elements in a single study, providing a powerful opportunity to triangulate, clarify and expand research findings [S79]. In general, there are three strategies for mixing both approaches [S80]. First, quantitative and qualitative data can be collected separately and brought together in the interpretation stage (known as ‘integration’). Second, findings from one approach can be used as the building base of the other (‘combination’); as can be seen in questionnaire development.^37^, ^45^ Third, the two data streams can be embedded so that a qualitative process evaluation is a fixed part within a quantitative clinical trial [S81].

### 5.2 Participatory approaches

Racial and ethnic minorities, lower income and rural communities and young adults are often underrepresented in studies, which means the findings we publish may not fully fit the populations who need them the most [S82, S83]. Emerging methodological approaches therefore need to move towards participatory designs that include the people whom the research is meant to support. For example, a recent study using stakeholder co‐creation workshops to address inequities in diabetes technology use demonstrates how participatory design can generate concrete, equity‐oriented solutions rather than top‐down plans [S84].

Looking ahead, we suggest that participation and equity should be design considerations rather than afterthoughts: plans for inclusive sampling and accessible materials, use structured involvement tools [S85] and report clearly on who was involved and how. Build capacity in teams and communities and budget for it. Finally, use participatory principles across qualitative, quantitative and mixed methods work: involve communities in setting questions and outcomes, co‐design interventions and measures and co‐evaluate them in samples that reflect those most affected [S82, S83, S86].

### 5.3 Realist evaluation

Whereas highly controlled evaluation studies (e.g. RCTs) determine effectiveness based on average quantitative effects, the underlying mechanisms are rarely investigated [S87, S88]. In contrast, realist evaluation seeks to answer ‘what works for whom, in what circumstances, how and why?’ for complex interventions [S89]. By focusing on within‐programme effects and combining qualitative and quantitative research methods, realist evaluations aim to understand how a program may generate different outcomes in different circumstances (contexts). As a realist focus can be applied across all phases of intervention development, evaluation and translation [S90], its approach is considered crucial in order to estimate the program's potential effects in different contexts and subgroups. For example, Garn et al. (2021) found that a peer support intervention improved self‐management and healthcare use only in participants with certain contextual factors, like stable occupation and optimal health [S91].

### 5.4 The power and pitfalls of AI

The use of AI for identifying, monitoring, predicting and treating mental health symptoms holds promise for improving the lives of people with diabetes. Recent reviews highlight the capabilities of AI to diagnose depression from behavioural cues (speech, texts, movement and facial expressions) [S92] and detect and predict depression from data from wearable devices [S57] in the general population. However, though encouraging, these methods are in their infancy, with research characterised by small sample sizes, methodological heterogeneity and variability in algorithms and devices used. This is not to mention the caution that must be exerted in relation to issues of privacy, data security, effectiveness and safety, a lack of legal frameworks and the potential for unforeseen consequences [S93] when considering the use of AI in clinical settings. As technology develops and AI algorithms are refined, collaboration between diabetes psychosocial researchers, computer scientists and AI specialists will be required to capitalise on this promise for use with people with diabetes.

## 6 CONCLUSION

Considering the rapid changes in diabetes therapy over the last years and the innovative therapy options on the horizon, it has never been more critical to apply novel qualitative and quantitative methodologies to understand the psychosocial impact of living with, managing and supporting people with diabetes. This can ensure that psychosocial diabetes research keeps pace with medical innovations and that the perspective and lived experiences of people with diabetes are adequately understood and addressed.

Novel statistical approaches, contextual insights, rich descriptions and narratives should aim at understanding the dynamics and intra‐ as well as inter‐person variability in diabetes experiences and offer the evidence for what, where, how and why. Current and emerging methodologies offer new opportunities to ask these questions in ways we never imagined possible, allowing us to develop and offer supports for people with diabetes to live their best life. Mixed methods evidence is a cornerstone for understanding the full scope of community needs and health care improvement opportunities.

Common to these approaches is the aim to dive deeper into the dynamics and intricacies of diabetes management of the individual in day‐to‐day life. Ultimately, these emerging qualitative and quantitative methodologies can progress the field of psychosocial diabetes research, ultimately moving beyond a group‐based ‘one‐size‐fits‐all’ approach to a precision medicine approach [S94] that entails a precision mental health approach [S95].

## AUTHOR CONTRIBUTIONS

D.E., E.L. and G.N. wrote the first draft of the article, with input from the other co‐authors. All authors contributed to the literature search and revised the manuscript for important intellectual content. All authors approved the final version of the article.

## FUNDING INFORMATION

No funding.

## CONFLICT OF INTEREST STATEMENT

The authors declare no conflict of interest with regard to the content of this manuscript. Authors span broad career stages, including PhD students, early‐ and mid‐career and senior researchers, with diverse, multidisciplinary backgrounds including behavioural psychology and medical/clinical as well as expertise across quantitative and qualitative research methods. Authors have dedicated their careers to improving the lives of people living with diabetes, all with “loved experience” of people living with type 1, type 2, gestational and or rarer types of diabetes.

## Supporting information


Data S1.

## Data Availability

Data sharing not applicable to this article as no datasets were generated or analysed during the current study.
