# Supplementary material for: From narratives to numbers and back: Assessing the psychosocial aspects of diabetes in the era of high technology with emerging qualitative and quantitative methodologies
Source: Diabet Med. 2026 Jan 2;43(9):e70206. doi: 10.1111/dme.70206 (PMC13613990; doi:10.1111/dme.70206)
Supplement: Supplementary file 1 [file DME-43-e70206-s001.docx]

**From narratives to numbers and back: Assessing the psychosocial aspects of diabetes in the era of high technology with emerging qualitative and quantitative methodologies**

Running title: Emerging methodologies in psychosocial diabetes research

Authors:

Dominic Ehrmann^1,2,3^, Eloise Litterbach^4,5^, Sonya Deschenes^6^, Rita Forde^7^, Norbert Hermanns^1,2,3^, Maaike Horsselenberg^8^, Mandy Jansen^8,9,10^, Amy McInerney^11^, Eimear Morrissey^12^, Andreas Schmitt^1,3^, Uffe Søholm^4,5^, Giesje Nefs^8,13,14,15^

Affiliations:

^1^ Research Institute Diabetes Academy Mergentheim (FIDAM), Bad Mergentheim, Germany

^2^ Department of Clinical Psychology and Psychotherapy, University of Bamberg, Bamberg, Germany

^3^ German Center for Diabetes Research (DZD), München-Neuherberg, Germany

^4^ School of Psychology | Institute for Health Transformation, Deakin University, Geelong, Australia

^5^ The Australian Centre for Behavioural Research in Diabetes, Diabetes Victoria, Carlton, Australia

^6^ School of Psychology, University College Dublin, Dublin, Ireland

^7^ School of Nursing and Midwifery, University College Cork, Ireland

^8^ Radboud university medical center, Department of Medical Psychology, Nijmegen, the Netherlands

^9^ CWZ Academy, Canisius-Wilhelmina Hospital, Nijmegen, The Netherlands

^10^ Center for Pediatric and Adolescent Diabetes, Vivendia, Nijmegen, The Netherlands

^11^ Department of Population-Based Medicine, University of Tuebingen, Germany

^12^ Centre for Health Research Methodology, School of Nursing and Midwifery, University of Galway, Ireland

^13^ Diabeter, Center for Focused Diabetes Care and Research, Rotterdam, the Netherlands;

^14^ Tilburg University, Department of Medical and Clinical Psychology, C*o*RPS – Center of Research on Psychological disorders and Somatic diseases, Tilburg, the Netherlands

^15^ Diabeter Centrum Amsterdam, Amsterdam, the Netherlands

**Supplementary references**

S1.	Delnoij DMJ, Derks M, Koolen L, Shekary S, Suitela J. Using Patient Blogs on Social Media to Assess the Content Validity of Patient-Reported Outcome Measures: Qualitative Analysis of Patient-Written Blogs. *JMIR Form Res* 2023; **7**:e43210.

S2.	Litterbach E, Holmes-Truscott E, Pouwer F, Speight J, Hendrieckx C. 'I wish my health professionals understood that it's not just all about your HbA(1c) !'. Qualitative responses from the second Diabetes MILES - Australia (MILES-2) study. *Diabet Med* 2020; **37**:971-981.

S3.	Kennedy EL, Gordon BA, Ng AH, Smith G, Forsyth AK. Barriers and enablers to health service access amongst people with diabetes: An exploration of the perceptions of health care staff in regional Australia. *Health Soc Care Community* 2022; **30**:e234-e244.

S4.	Sibounheuang P, Olson PS, Kittiboonyakun P. Patients' and healthcare providers’ perspectives on diabetes management: A systematic review of qualitative studies. *Research in Social and Administrative Pharmacy* 2020; **16**:854-874.

S5.	Litterbach E, Holmes-Truscott E, Gray S, Halliday J, Scibilia R, Skinner T*, et al.* “I feel like I'm being talked to like an equal”: Diabetes language matters to adults with diabetes, a mixed-methods study. *Diabetic Medicine* 2024; **41**:e15424.

S6.	Adu MD, Malabu UH, Malau-Aduli AEO, Malau-Aduli BS. Enablers and barriers to effective diabetes self-management: A multi-national investigation. *PLoS One* 2019; **14**:e0217771.

S7.	Gucciardi E, Reynolds E, Karam G, Beanlands H, Sidani S, Espin S. Group-based storytelling in disease self-management among people with diabetes. *Chronic Illn* 2021; **17**:306-320.

S8.	Pong CY, Roberts NJ, Lum E. The “what, why, and how?” of story completion in health services research: a scoping review. *BMC Medical Research Methodology* 2024; **24**:159.

S9.	Geia LK, Hayes B, Usher K. Yarning/Aboriginal storytelling: Towards an understanding of an Indigenous perspective and its implications for research practice. *Contemporary Nurse* 2013; **46**:13-17.

S10.	Lin I, Flanagan W, Green C, Lowell A, Coffin J, Bessarab D. Clinical yarning education: development and pilot evaluation of an education program to improve clinical communication in Aboriginal health care - participant, and health manager perspectives. *BMC Med Educ* 2023; **23**:908.

S11.	Omodei-James S, Wilson A, Kropinyeri R, Cameron D, Wingard S, Kerrigan C*, et al.* Exploration of barriers and enablers to diabetes care for Aboriginal people on rural Ngarrindjeri Country. *Health Promot J Austr* 2025; **36**:e915.

S12.	Kennedy M, Maddox R, Booth K, Maidment S, Chamberlain C, Bessarab D. Decolonising qualitative research with respectful, reciprocal, and responsible research practice: a narrative review of the application of Yarning method in qualitative Aboriginal and Torres Strait Islander health research. *International Journal for Equity in Health* 2022; **21**:134.

S13.	Walker M, Fredericks B, Mills K, Anderson D. “Yarning” as a Method for Community-Based Health Research With Indigenous Women: The Indigenous Women's Wellness Research Program. *Health Care for Women International* 2014; **35**:1216-1226.

S14.	Thunberg S, Arnell L. Pioneering the use of technologies in qualitative research – A research review of the use of digital interviews. *International Journal of Social Research Methodology* 2022; **25**:757-768.

S15.	Chatwin H, Broadley M, Hendrieckx C, Carlton J, Heller S, Amiel SA*, et al.* Unmet support needs relating to hypoglycaemia among adults with type 1 diabetes: Results of a multi-country web-based qualitative study. *Diabetic Medicine* 2022; **39**:e14727.

S16.	Chatwin H, Broadley M, Valdersdorf Jensen M, Hendrieckx C, Carlton J, Heller S*, et al.* 'Never again will I be carefree': a qualitative study of the impact of hypoglycemia on quality of life among adults with type 1 diabetes. *BMJ Open Diabetes Research & Care* 2021; **9**:e002322.

S17.	Goyanes M, Lopezosa C, Jordá B. Thematic analysis of interview data with ChatGPT: designing and testing a reliable research protocol for qualitative research. *Quality & Quantity* 2025.

S18.	Tang L, Gu J, Lin Z. A scoping review of photovoice for people living with diabetes. *BMC Public Health* 2025; **25**:540.

S19.	Nykiforuk CIJ. Engaging patients in research using photovoice methodology. *Canadian Medical Association Journal* 2021; **193**:E1050.

S20.	Tariq S, Reed T, Auger J, Bowdridge B, Larsen M, Lawson J*, et al.* Illustrating stories of stigma from the perspective of people living with diabetes while experiencing homelessness: An arts-based community participatory research project. *Diabetic Medicine* 2025; **n/a**:e70113.

S21.	Liesch SK, Elertson KM. Drawing and Dialogue: Youth's Experiences With the "Face" of Diabetes. *J Patient Exp* 2020; **7**:1158-1163.

S22.	Chalmers K, Smith M, Moreno M, Malik F. “It Got Likes, But I Don’t Think People Understood”: A Qualitative Study of Adolescent Experiences Discussing Type 1 Diabetes on Social Media. *Journal of Diabetes Science and Technology* 2020; **16**:858-865.

S23.	Arillotta D, Floresta G, Guirguis A, Corkery JM, Catalani V, Martinotti G*, et al.* GLP-1 Receptor Agonists and Related Mental Health Issues; Insights from a Range of Social Media Platforms Using a Mixed-Methods Approach. *Brain Sciences* 2023.

S24.	Maxwell T, Branka L, Asher N, Commissariat P, Laffel L. Young Adults With Type 1 Diabetes and Their Perspectives on Diabetes-Related Social Media: Qualitative Study. *JMIR Diabetes* 2025; **10**:e69243.

S25.	Barbour K, Lee K, Moore C. Online persona research: An Instagram case study. 2017: 1-12.

S26.	Staite E, Zaremba N, Macdonald P, Allan J, Treasure J, Ismail K*, et al.* ‘Diabulima’ through the lens of social media: a qualitative review and analysis of online blogs by people with Type 1 diabetes mellitus and eating disorders. *Diabetic Medicine* 2018; **35**:1329-1336.

S27.	Massey PM, Murray RM, Chiang SC, Russell AM, Yudell MA. Social Media, Public Health Research, and Vulnerability: Considerations to Advance Ethical Guidelines and Strengthen Future Research. *JMIR Public Health Surveill* 2023; **9**:e49881.

S28.	Sushko K, Strachan P, Butt M, Nerenberg KA, Sherifali D. Understanding the self-management experiences and support needs during pregnancy among women with pre-existing diabetes: a qualitative descriptive study. *BMC pregnancy and childbirth* 2023; **23**:309.

S29.	Barnard K, Skinner TC. Qualitative study into quality of life issues surrounding insulin pump use in type 1 diabetes. *Practical Diabetes International* 2007; **24**:143-148.

S30.	Hortensius J, Kars MC, Wierenga WS, Kleefstra N, Bilo HJ, van der Bijl JJ. Perspectives of patients with type 1 or insulin-treated type 2 diabetes on self-monitoring of blood glucose: a qualitative study. *BMC Public Health* 2012; **12**:167.

S31.	Holmes-Truscott E, Hateley-Browne JL, Charalambakis E, Ventura AD, Ripper A, Scibilia R*, et al.* Diabetes misconceptions, seriousness, motivation, self-efficacy and stigma: A cross-sectional comparison of eight Australian diabetes communication campaign videos. *Diabetic Medicine* 2024; **41**:e15399.

S32.	Hunt D, Lamb K, Elliott J, Hemmingsen B, Slama S, Scibilia R*, et al.* A WHO key informant language survey of people with lived experiences of diabetes: Media misconceptions, values-based messaging, stigma, framings and communications considerations. *Diabetes Research and Clinical Practice* 2022; **193**.

S33.	Battelino T, Danne T, Bergenstal RM, Amiel SA, Beck R, Biester T*, et al.* Clinical Targets for Continuous Glucose Monitoring Data Interpretation: Recommendations From the International Consensus on Time in Range. *Diabetes Care* 2019; **42**:1593-1603.

S34.	Luo X, Ma J, Hu Y. A dynamic bidirectional system of stress processes: Feedback loops between stressors, psychological distress, and physical symptoms. *Health Psychol* 2025; **44**:154-165.

S35.	Wichers M. The dynamic nature of depression: a new micro-level perspective of mental disorder that meets current challenges. *Psychol Med* 2014; **44**:1349-1360.

S36.	Borsboom D. A network theory of mental disorders. *World Psychiatry* 2017; **16**:5-13.

S37.	Nam S, Griggs S, Ash GI, Dunton GF, Huang S, Batten J*, et al.* Ecological momentary assessment for health behaviors and contextual factors in persons with diabetes: A systematic review. *Diabetes Res Clin Pract* 2021; **174**:108745.

S38.	Ehrmann D, Schmitt A, Priesterroth L, Kulzer B, Haak T, Hermanns N. Time With Diabetes Distress and Glycemia-Specific Distress: New Patient-Reported Outcome Measures for the Psychosocial Burden of Diabetes Using Ecological Momentary Assessment in an Observational Study. *Diabetes Care* 2022; **45**:1522-1531.

S39.	Ehrmann D, Priesterroth L, Schmitt A, Kulzer B, Hermanns N. Associations of Time in Range and Other Continuous Glucose Monitoring-Derived Metrics With Well-Being and Patient-Reported Outcomes: Overview and Trends. *Diabetes Spectr* 2021; **34**:149-155.

S40.	Hermanns N, Ehrmann D, Shapira A, Kulzer B, Schmitt A, Laffel L. Coordination of glucose monitoring, self-care behaviour and mental health: achieving precision monitoring in diabetes. *Diabetologia* 2022; **65**:1883-1894.

S41.	Polonsky WH, Fortmann AL. The influence of time in range on daily mood in adults with type 1 diabetes. *J Diabetes Complications* 2020; **34**:107746.

S42.	Jin H, Gonzalez JS, Pyatak EA, Schneider S, Hoogendoorn CJ, Hernandez R*, et al.* Within-person relationships of sleep duration with next-day stress and affect in the daily life of adults with Type-1 diabetes. *J Psychosom Res* 2023; **173**:111442.

S43.	Pyatak EA, Spruijt-Metz D, Schneider S, Hernandez R, Pham LT, Hoogendoorn CJ*, et al.* Impact of Overnight Glucose on Next-Day Functioning in Adults With Type 1 Diabetes: An Exploratory Intensive Longitudinal Study. *Diabetes Care* 2023; **46**:1345-1353.

S44.	Shapira A, Volkening LK, Borus JS, Laffel LM. Ecological Momentary Assessment of Positive and Negative Affect and Associations with Blood Glucose in Teens with Type 1 Diabetes. *J Diabetes Sci Technol* 2023; **17**:195-200.

S45.	Hermanns N, Ehrmann D, Kulzer B, Klinker L, Haak T, Schmitt A. Somatic and mental symptoms associated with dysglycaemia, diabetes-related complications and mental conditions in people with diabetes: Assessments in daily life using continuous glucose monitoring and ecological momentary assessment. *Diabetes Obes Metab* 2025; **27**:61-70.

S46.	Søholm U, Broadley M, Zaremba N, Divilly P, Baumann PM, Mahmoudi Z*, et al.* The impact of hypoglycaemia on daily functioning among adults with diabetes: a prospective observational study using the Hypo-METRICS app. *Diabetologia* 2024; **67**:2160-2174.

S47.	Zuniga-Kennedy M, Wang OH, Fonseca LM, Cleveland MJ, Bulger JD, Grinspoon E*, et al.* Nocturnal hypoglycemia is associated with next day cognitive performance in adults with type 1 diabetes: Pilot data from the GluCog study. *Clin Neuropsychol* 2024; **38**:1627-1646.

S48.	Ehrmann D, Hermanns N, Schmitt A, Klinker L, Haak T, Kulzer B. Perceived glucose levels matter more than CGM-based data in predicting diabetes distress in type 1 or type 2 diabetes: a precision mental health approach using n-of-1 analyses. *Diabetologia* 2024; **67**:2433-2445.

S49.	Bolger N, Laurenceau J-P. Intensive longitudinal methods: An introduction to diary and experience sampling research: Guilford Press; 2013.

S50.	Pyatak EA, Hernandez R, Pham LT, Mehdiyeva K, Schneider S, Peters A*, et al.* Function and Emotion in Everyday Life With Type 1 Diabetes (FEEL-T1D): Protocol for a Fully Remote Intensive Longitudinal Study. *JMIR Res Protoc* 2021; **10**:e30901.

S51.	Søholm U, Broadley M, Zaremba N, Divilly P, Nefs G, Mahmoudi Z*, et al.* Investigating the day-to-day impact of hypoglycaemia in adults with type 1 or type 2 diabetes: design and validation protocol of the Hypo-METRICS application. *BMJ Open* 2022; **12**:e051651.

S52.	Onnela JP. Opportunities and challenges in the collection and analysis of digital phenotyping data. *Neuropsychopharmacology* 2021; **46**:45-54.

S53.	Pai A, Santiago R, Glantz N, Bevier W, Barua S, Sabharwal A*, et al.* Multimodal digital phenotyping of diet, physical activity, and glycemia in Hispanic/Latino adults with or at risk of type 2 diabetes. *NPJ Digit Med* 2024; **7**:7.

S54.	Knauer J, Baumeister H, Schmitt A, Terhorst Y. Acceptance of smart sensing, its determinants, and the efficacy of an acceptance-facilitating intervention in people with diabetes: results from a randomized controlled trial. *Front Digit Health* 2024; **6**:1352762.

S55.	McInerney AM, Schmitz N, Matthews M, Deschênes SS. “Anything that would help is a positive development”: feasibility, tolerability, and user experience of smartphone-based digital phenotyping for people with and without type 2 diabetes. *BMC Digital Health* 2024; **2**:55.

S56.	Aguirre Vergara F, Pinker I, Fischer A, Seuring T, Tichomirowa MA, de Beaufort C*, et al.* Readiness of adults with type 1 diabetes and diabetes caregivers for diabetes distress monitoring using a voice-based digital health solution: insights from the PsyVoice mixed methods study. *BMJ Open* 2025; **15**:e088424.

S57.	Abd-Alrazaq A, AlSaad R, Shuweihdi F, Ahmed A, Aziz S, Sheikh J. Systematic review and meta-analysis of performance of wearable artificial intelligence in detecting and predicting depression. *NPJ Digit Med* 2023; **6**:84.

S58.	Ahlqvist E, Storm P, Karajamaki A, Martinell M, Dorkhan M, Carlsson A*, et al.* Novel subgroups of adult-onset diabetes and their association with outcomes: a data-driven cluster analysis of six variables. *Lancet Diabetes Endocrinol* 2018; **6**:361-369.

S59.	Reutterer T, Dan D. Cluster Analysis in Marketing Research. In: Homburg C, M Klarmann, A Vomberg, eds. *Handbook of Market Research*. Cham: Springer International Publishing 2019: 1-29.

S60.	Kubiak T, Priesterroth L, Barnard-Kelly KD. Psychosocial aspects of diabetes technology. *Diabet Med* 2020; **37**:448-454.

S61.	Clatworthy J, Buick D, Hankins M, Weinman J, Horne R. The use and reporting of cluster analysis in health psychology: a review. *Br J Health Psychol* 2005; **10**:329-358.

S62.	Weller BE, Bowen NK, Faubert SJ. Latent Class Analysis: A Guide to Best Practice. *Journal of Black Psychology* 2020; **46**:287-311.

S63.	Tanenbaum ML, Adams RN, Iturralde E, Hanes SJ, Barley RC, Naranjo D*, et al.* From Wary Wearers to d-Embracers: Personas of Readiness to Use Diabetes Devices. *Journal of Diabetes Science and Technology* 2018; **12**:1101-1107.

S64.	Wong JJ, Barley RC, Hanes S, Tanenbaum ML, Lanning M, Naranjo D*, et al.* Parental Perspectives: Identifying Profiles of Parental Attitudes and Barriers Related to Diabetes Device Use. *Diabetes Technol Ther* 2020; **22**:674-680.

S65.	Tanenbaum ML, Adams RN, Lanning MS, Hanes SJ, Agustin BI, Naranjo D*, et al.* Using Cluster Analysis to Understand Clinician Readiness to Promote Continuous Glucose Monitoring Adoption. *J Diabetes Sci Technol* 2018; **12**:1108-1115.

S66.	Heron KE, Smyth JM. Ecological momentary interventions: incorporating mobile technology into psychosocial and health behaviour treatments. *Br J Health Psychol* 2010; **15**:1-39.

S67.	Nahum-Shani I, Smith SN, Spring BJ, Collins LM, Witkiewitz K, Tewari A*, et al.* Just-in-Time Adaptive Interventions (JITAIs) in Mobile Health: Key Components and Design Principles for Ongoing Health Behavior Support. *Ann Behav Med* 2018; **52**:446-462.

S68.	Wang L, Miller LC. Just-in-the-Moment Adaptive Interventions (JITAI): A Meta-Analytical Review. *Health Commun* 2020; **35**:1531-1544.

S69.	Schneider S, Junghaenel DU, Smyth JM, Fred Wen CK, Stone AA. Just-in-time adaptive ecological momentary assessment (JITA-EMA). *Behav Res Methods* 2024; **56**:765-783.

S70.	Cappon G, Facchinetti A. Digital Twins in Type 1 Diabetes: A Systematic Review. *J Diabetes Sci Technol* 2024; **0**:19322968241262112.

S71.	Mosquera-Lopez C, Jacobs PG. Digital twins and artificial intelligence in metabolic disease research. *Trends Endocrinol Metab* 2024; **35**:549-557.

S72.	Fagherazzi G. Deep Digital Phenotyping and Digital Twins for Precision Health: Time to Dig Deeper. *J Med Internet Res* 2020; **22**:e16770.

S73.	Ehrmann D, Kulzer B, Schmitt A, Klinker L, Hermanns N. Künstliche Intelligenz als „decision support system“. *Die Diabetologie* 2025.

S74.	Walton A, Nahum-Shani I, Crosby L, Klasnja P, Murphy S. Optimizing Digital Integrated Care via Micro-Randomized Trials. *Clinical Pharmacology & Therapeutics* 2018; **104**:53-58.

S75.	Kidwell KM, Almirall D. Sequential, Multiple Assignment, Randomized Trial Designs. *JAMA* 2023; **329**:336-337.

S76.	Lonsdorf TB, Gerlicher A, Klingelhöfer-Jens M, Krypotos A-M. Multiverse analyses in fear conditioning research. *Behaviour Research and Therapy* 2022; **153**:104072.

S77.	Rengasamy M, Moriarity D, Kraynak T, Tervo-Clemmens B, Price R. Exploring the multiverse: the impact of researchers’ analytic decisions on relationships between depression and inflammatory markers. *Neuropsychopharmacology* 2023; **48**:1465-1474.

S78.	Niemeijer K, Mestdagh M, Kuppens P. Tracking Subjective Sleep Quality and Mood With Mobile Sensing: Multiverse Study. *J Med Internet Res* 2022; **24**:e25643.

S79.	Halcomb EJ. Mixed methods research: The issues beyond combining methods. *Journal of Advanced Nursing* 2019; **75**:499-501.

S80.	Zhang W, Creswell J. The use of "mixing" procedure of mixed methods in health services research. *Med Care* 2013; **51**:e51-57.

S81.	Papachristou Nadal I, Aramrat C, Jiraporncharoen W, Pinyopornpanish K, Wiwatkunupakarn N, Quansri O*, et al.* Process evaluation protocol of a cluster randomised trial for a scalable solution for delivery of Diabetes Self-Management Education in Thailand (DSME-T). *BMJ Open* 2021; **11**:e056141.

S82.	Ahmed R, de Souza RJ, Li V, Banfield L, Anand SS. Twenty years of participation of racialised groups in type 2 diabetes randomised clinical trials: a meta-epidemiological review. *Diabetologia* 2024; **67**:443-458.

S83.	Akturk HK, Agarwal S, Hoffecker L, Shah VN. Inequity in Racial-Ethnic Representation in Randomized Controlled Trials of Diabetes Technologies in Type 1 Diabetes: Critical Need for New Standards. *Diabetes Care* 2021; **44**:e121-e123.

S84.	Agarwal S, Crespo-Ramos G, Leung SL, Finnan M, Park T, McCurdy K*, et al.* Solutions to Address Inequity in Diabetes Technology Use in Type 1 Diabetes: Results from Multidisciplinary Stakeholder Co-creation Workshops. *Diabetes Technol Ther* 2022; **24**:381-389.

S85.	Smits D-W, van Meeteren K, Klem M, Alsem M, Ketelaar M. Designing a tool to support patient and public involvement in research projects: the Involvement Matrix. *Research Involvement and Engagement* 2020; **6**:30.

S86.	Zoltick ES, Wu AC, Ong M-S. Racial and ethnic representation of youth in type 1 diabetes interventional trials. *Contemporary Clinical Trials* 2024; **146**:107703.

S87.	Chen HT, Garbe P. Assessing program outcomes from the bottom-up approach: An innovative perspective to outcome evaluation. *New Directions for Evaluation* 2011; **2011**:93-106.

S88.	Pawson R, Tilley N. Realistic Evaluation. London: SAGE Publications 1997.

S89.	Wong G, Westhorp G, Greenhalgh J, Manzano A, Jagosh J, Greenhalgh T. Health Services and Delivery Research. *Quality and reporting standards, resources, training materials and information for realist evaluation: the RAMESES II project*. Southampton (UK): NIHR Journals Library 2017.

S90.	Fletcher A, Jamal F, Moore G, Evans RE, Murphy S, Bonell C. Realist complex intervention science: Applying realist principles across all phases of the Medical Research Council framework for developing and evaluating complex interventions. *Evaluation (Lond)* 2016; **22**:286-303.

S91.	Garn SD, Glümer C, Villadsen SF, Malling GMH, Christensen U. Understanding the mechanisms generating outcomes in a Danish peer support intervention for socially vulnerable people with type 2-diabetes: a realist evaluation. *Arch Public Health* 2021; **79**:160.

S92.	Goh YS, See QR, Vongsirimas N, Klanin-Yobas P. Artificial Intelligence in Diagnosing Depression Through Behavioural Cues: A Diagnostic Accuracy Systematic Review and Meta-Analysis. *Journal of Clinical Nursing* 2025; **n/a**.

S93.	Zafar F, Fakhare Alam L, Vivas RR, Wang J, Whei SJ, Mehmood S*, et al.* The Role of Artificial Intelligence in Identifying Depression and Anxiety: A Comprehensive Literature Review. *Cureus* 2024; **16**:e56472.

S94.	Chung WK, Erion K, Florez JC, Hattersley AT, Hivert MF, Lee CG*, et al.* Precision medicine in diabetes: a Consensus Report from the American Diabetes Association (ADA) and the European Association for the Study of Diabetes (EASD). *Diabetologia* 2020; **63**:1671-1693.

S95.	Pouwer F, Barnard-Kelly K, Cleal BR, Cooke D, de Groot M, Deschênes S*, et al.* The missing piece: The clinical translation of precision diabetes medicine requires precision mental health care: A call to action from the international PsychoSocial Aspects of Diabetes (PSAD) Study Group. *Diabetic Medicine* 2025; **42**.
